# Current recycling innovations to utilize e-waste in sustainable green metal manufacturing

**DOI:** 10.1098/rsta.2023.0239

**Published:** 2024-11-04

**Authors:** Rumana Hossain, Veena Sahajwalla

**Affiliations:** ^1^ Centre for Sustainable Materials Research and Technology, SMaRT@UNSW, School of Materials Science and Engineering, UNSW Sydney, Sydney, New South Wales, Australia

**Keywords:** sustainable metal manufacturing, electronic waste, recycling, waste management, circular economy

## Abstract

The ever-increasing market demand and the rapid uptake of the technologies of electronics create an unavoidable generation of high-volume electronic waste (e-waste). E-waste is embedded with valuable metals, alloys, precious metals and rare earth elements. A substantial portion of e-waste ends up in landfills and is incinerated due to its complex multi-material structure, creating loss of resources and often leading to environmental contamination from the release of landfill leachates and combustion gases. Conversely, due to the ongoing demand for valuable metals, global industrial and manufacturing supply chains are experiencing enormous pressure. To address this issue, researchers have put multifaceted efforts into developing viable technologies and emphasized right-scaling for e-waste reclamation. Several conventional and emerging recycling technologies have been recognized to be efficient in recovering metal alloys, precious and rare earth metals from e-waste. The recovery of valuable metals from e-waste will create an alternative source of value-added raw materials, which could become part of supply chains for manufacturing. This review discusses the urgency of metal recycling from e-waste for sustainability and economic benefit, up-to-date recycling technologies with an emphasis on their potential role in creating a circular economy in e-waste management.

This article is part of the discussion meeting issue ‘Sustainable metals: science and systems’.

## 1. Introduction

The global demand for consumer electronics like handheld devices, laptops, computers and kitchen appliances, and the widespread adoption of electric vehicles has led to the exponential increase in the generation of electrical and electronics waste (e-waste) [1]. The annual e-waste generation reached more than 62 million tonnes per year in 2022 [2], making it the fastest growing waste stream globally. This massive volume, containing potentially hazardous materials like heavy metals and organic chemicals, poses significant environmental threats [3,4]. Consequently, the implementation of efficient and effective e-waste management strategies is imperative to mitigate these detrimental environmental impacts.

However, a substantial portion of e-waste is still managed through traditional methods such as landfilling and incineration in developing countries [5], while developed countries are transporting their waste to less privileged countries (sometimes illegally) prioritizing volume reduction [6]. These practices, while simple in implementation, incur significant environmental consequences, including the emission of harmful gas into the atmosphere, the generation of contaminated wastewater and soil pollution [6]. Moreover, the retrofitting of existing treatment facilities with additional pollution control equipment exacerbates waste management costs, potentially hindering the financial viability and widespread accessibility of these approaches [3,7].

Despite containing valuable inorganic components, including rare earth elements (REEs) such as lanthanum (La), neodymium (Nd) and dysprosium (Dy), precious metals like platinum (Pt), gold (Au) and silver (Ag), and other valuable metals like lithium (Li), nickel (Ni) and titanium (Ti), only 22.3% of these metals are possibly recovered as per the recycling rate of 2022 [8]. This results in an annual loss of approximately USD 62 billion worth of metals through landfilling, incineration and other improper methods [2]. To address this challenge, researchers are increasingly focusing on the development of circular economy platforms for e-waste, primarily through technical enhancements [3,9]. Notably, various metallurgical processes, such as hydrometallurgy, biometallurgy, pyrometallurgy and hybrid approaches, are being explored to achieve more efficient metal recovery from e-waste. As metals constitute critical resources for diverse industries, their sustainable extraction from e-waste offers economic benefits by directly reducing supply costs within these sectors [10].

Therefore, this review was prepared to provide a comprehensive overview of recovering the valuable metals and alloys from e-waste. Recycling of metals from the e-waste that is embedded with valuable metallic compounds, such as printed circuit boards (PCBs), small consumer electronics, hard drives and batteries, are considered in this work. The sources and the value of the embedded resources, their recycling technological intervention and circular economy of the materials for these e-waste metals, are discussed.

## 2. Global status of e-waste

### (a) Definition and classification of e-waste

The United States Environmental Protection Agency (USEPA) defines e-waste as ‘any electronic device that has reached the end of its useful life and is no longer wanted for its original purpose’ [11]. This broad definition, also known as Waste Electrical and Electronics Equipment (WEEE) or electronic scrap, can lead to confusion. In an attempt to manage this ambiguity, numerous countries have created their own classification systems. However, achieving a globally standardized approach remains elusive, resulting in diverse classification schemes across various regions.

The European Union (EU) has adopted a six-category system for e-waste: (1) temperature-exchange equipment, (2) screens and monitors, (3) lamps, (4) large equipment (dimensions > 50 cm), (5) small equipment (dimensions ≤ 50 cm) and (6) small IT and telecommunication equipment (dimensions ≤ 50 cm). By contrast, China relies on a 10-category structure: (1) refrigerators, (2) monitors and TVs, (3) washing machines, (4) air conditioners, (5) water heaters, (6) personal computers, (7) fax and telephone machines, (8) mobile phones, (9) printers and (10) copiers [12]. The United Nations University’s (UNU-Keys) classification system offers a granular approach to e-waste categorization, dividing it into 54 distinct categories encompassing diverse items like central heating units, photovoltaic panels, dishwashers and washing machines [13]. By contrast, both India and Japan adopt simple classification schemes. India categorizes e-waste into two broad groups: (1) information technology and telecommunication equipment (IT&TE) comprising computers, laptops, phones and printers, and (2) consumer electrical and electronics (CEE) including televisions, refrigerators and washing machines [14]. Similarly, Japan divides e-waste into two categories: (1) large home appliances like air conditioners and refrigerators, and (2) small home appliances such as PCs, mobile phones and digital cameras [15].

### (b) E-waste production

The generation of e-waste jumped from 41.8 million metric tonnes in 2014 to 53.6 million metric tonnes in 2019, reflecting a significant upsurge [16]. This trend persists on a per capita basis, as depicted in global per capita e-waste production, which rose from 6.4 kg/person in 2014 [16] to 7.8 kg/person in 2023 [17], indicating widespread growth. Analysing the waste category and classification composition of e-waste reveals that large-scale equipment, small-scale equipment and temperature control equipment hold the top three positions, respectively. Notably, temperature control equipment (20.1%), large equipment (24.4%), electric lights (20.1%) and small equipment (32.5%) exhibit the fastest growth rates, while small electronic communication equipment experiences a slower increase (8.8%) [16]. As of 2019, only 17.4% of e-waste was officially recycled, leaving the fate of the remaining 82.6% ambiguous. Geographically, Asia leads in e-waste production, generating approximately 24.9 million metric tonnes in 2019. America and Europe follow with 13.1 and 12 million metric tonnes, respectively, while Africa and Oceania contribute considerably less, at 2.9 million and 7 00 000 metric tonnes, respectively [16]. In 2019, Europe led the world in e-waste production per capita, at 16.2 kg, followed closely by Oceania (16.1 kg) and America (13.3 kg) [16]. Asia and Africa demonstrate significantly lower per capita production, at 5.6 and 2.5 kg, respectively. These data suggest that e-waste generation in Europe and Oceania far exceeds those of other continents on a per-person basis.

### (c) Metal composition in e-waste

The metallic composition in electronic devices is influenced by factors such as device type, age and manufacturer [18]. Electronic supplementary material, table S1, provides a comprehensive overview of the metal composition found across diverse components within these devices. Notably, e-waste constitutes a valuable reservoir of reclaimable metals, including gold (Au), silver (Ag), copper (Cu), iron (Fe) and REEs [3]. Among these, copper holds the greatest abundance in electronic devices, followed closely by aluminium and iron, as clearly identified in table 1. Furthermore, in the electronic supplementary material, table S1 indicates that e-waste, such as hard drives, contain REEs, classified as Critical Raw Materials by the EU and as Critical Mineral Resources by the United States Department of the Interior [20] . These reclaimed metals find diverse applications across various industries, including optical/magnetic/chemical engineering, high-temperature superconductors, laser materials, secondary batteries and luminescence [21–24].

**Table 1.** Annual market price of the metals, REEs and precious metals found in e-waste (2014 to 2023) [19].

year	annual market price/tonne (USD)							
	Li	Ni	Co	Cu	Al	Fe	Zn	Sn
2014		1595.7	32 670.1	7257.6	2066.8	95.8	2309.8	22 921.9
2015		13 564.2	31 158.7	5600.0	1705.3	57.3	1947.1	15 440.8
2016		8652.4	24 357.7	4972.8	1652.4	61.2	2027.7	17 934.5
2017	19 592.9	10 289.7	57 858.9	6451.2	1943.3	72.1	2783.4	20 201.5
2018	16 164.1	13 186.4	76 246.9	6316.8	2066.8	68.2	2652.4	20 314.9
2019	9796.4	12 556.7	26 876.9	6160.0	1731.7	112.2	2430.7	18 501.9
2020	5388.1	12 682.6	29 143.9	6496.0	1625.9	113.4	2138.5	16 687.7
2021	12 490.5	16 083.1	50 806.1	9744.0	2631.0	188.7	3015.1	31 649.9
2022	66 126.2	2804.9	55 591.9	7929.6	2516.4	107.8	3246.8	25 075.6
2023	42 614.6	23 639.8	32 921.9	8825.6	2287.2	115.6	2450.9	27 796.0

year	annual market price/kg (USD)							
	Nd	Dy	La	In	Er	Au	Ag	
2014	59.6					59 652.1	947.3	
2015	49.4					50 861.6	682.8	
2016	46.6		1.9		26.4	62 373.1	934.7	
2017	54.0					57 504.6	758.0	
2018	59.6	238.1	1.9	380.1	24.6	53 484.0	682.4	
2019	56.2	238.1	8.8	428.6	24.0	61 627.6	706.8	
2020	57.3	345.2	6.4	315.1	23.0	84 091.4	1032.9	
2021	92.9	411.1	6.4	315.1	24.0	72 066.0	1034.7	
2022	156.6	769.6	6.7	477.1	21.0	65 904.4	759.4	
2023	83.9	653.4	6.5	442.3	18.0	71 035.6	894.2	
2024	69.8	587.4	6.3	561.6	17.0	72 845.2	805.0	

### (d) Market price of e-waste

The relentless expansion of the electronic industry has ignited a corresponding escalation in global demand for metals crucial to device manufacturing. Supply bottlenecks and trade restrictions along with the discrepancy in supply and demand are resulting in a significant rise in their prices. Reports indicate that the global market size for Cu and Au reached staggering values of more than USD 304 billion [25] and USD 198 billion [26], respectively, in 2022. This is because the demand for Cu is increasing abruptly because of the underlying demand in different industries and as energy transition demand continues to escalate. Demand for Au is increasing because of various factors, such as diversifying the asset base, and fuel price, and the production of Au has plateaued over the last few decades. Table 1 presents the annual metal prices (Li, Ni, Co, Cu, Al, Fe, Au, Er) from 2014 to 2023, revealing a general upward trend for most metals, particularly the critical metals, albeit with a temporary decline in 2019–2020 attributable to the disruptions caused by the COVID-19 pandemic [27]. There are a multitude of other reasons for the price fluctuations, such as unstable and tense geopolitical situations, increasing commodity prices disrupted and tightened supply chain, etc. These escalating demands coupled with fluctuating prices underscore the critical need for a feasible and efficient metal recovery process to safeguard the stability of the metal supply chain.

Furthermore, the current metal supply chain within the electronics industry poses substantial economic and environmental burdens due to its heavy resilience on coal consumption and the generation of wastewater requiring extensive post-treatment processes [9]. For instance, the extraction of 1 tonne of Cu from copper pre-demands a staggering 1400–1700 kg of standard coal for energy, simultaneously producing 20 tonnes of wastewater and 3 tonnes of hazardous solid waste, as per Chinese national standards for Cu smelting [28,29]. Extraction of 1 tonne Li needs 250 tonnes of spodumene or 750 tonnes of mineral-rich brine [30,31], which decreases the water level directly [32]. Besides, several thermochemical processes, such as selective thermal transformation, pyrolysis and catalytic pyrolysis, have been utilized to recycle plastic materials in e-waste and recover them as carbons, oils and gases (electronic supplementary material, table S2) into fuel gases and other value-added chemicals.

## 3. E-waste recycling strategies for metal extraction

### (a) Pretreatment

E-waste, a mix of metallic and non-metallic compounds, demands specific target-oriented recycling processes. To optimize recovery and minimize energy consumption, pretreatment processes like crushing and separation are crucial. For the pretreatment, the initial stage involves sorting and dismantling, breaking down the waste into electronics, fasteners and other components for reusable part separation. Subsequently, after separating the reusable parts, the scrap e-waste is shredded and screened for size reduction. Shredded particles then undergo a separation process for target metals: magnetic separation for ferrous metals, for example, and eddy current for non-ferrous metals and non-metals.

Finally, metallic and non-metallic compounds are separated using density-based methods, where embedded metallic components from the non-metallic framework are separated using an additional metal extraction process for high-purity metals [33–36]. Hydrometallurgy, biometallurgy, pyrometallurgy and hybrid metallurgy are explored and implemented for this purpose. The selection of the extraction process considers factors like target metals, separation efficiency, environmental impact and cost. The working principles and relevant applications of each metallurgical method are discussed below.

### (b) Hydrometallurgy

Traditional hydrometallurgy is a widely employed and highly efficient method for extracting metals from e-waste [37,38] that relies on chemical dissolution using acids (H_2_SO_4_, HNO_3_, C_2_H_4_O_2_, C_2_H_2_O_4_) and oxidants ([NH_4_]_2_S_2_O_8_ and H_2_O_2_) [37]. However, this conventional approach necessitates substantial chemical consumption, leading to secondary pollution and increased financial burden [3]. Also, the effectiveness of hydrometallurgy depends a lot on what kind of e-waste fraction is treated, the target metals and the degree of pretreatment.

There have been emerging alternatives to conventional hydrometallurgy that are described in table 2:

- Electrochemical methods: electrochemical methods are not novel for the recovery of non-ferrous methods. However, it could be beneficial to incorporate their application with hydrometallurgy methods. By utilizing electrical currents for selective precipitation of target metals, these methods minimize the need for additional chemicals during the precipitation step and even generate valuable by-products like hydroxyl groups and H_2_ [92]. For instance, Yi *et al.* achieved a remarkable 95% Cu recovery from shredded CPUs using an electrolysis cell filled with NaCl, HCl and H_2_O_2_ electrolyte [48]. Chan *et al.* further demonstrated the potential of this approach by recovering Li, Ni and Co from lithium-ion phosphate batteries with efficiencies of 99.0%, 99.3% and 87.3%, respectively [50].
- Ionic liquid extractions (ILE): this approach employs designer solvents like ionic liquids and deep eutectic solvents, such as trihexyl(tetradecyl)phosphonium nitrate (C_32_H_68_NO_3_P), trihexyl(tetradecyl)phosphonium thiocyanate (C_33_H_68_NPS), trimethyltrioctyl/decylammonium bis(2,4,4-trimethylpentyl)phosphate (R4NCy), C_25_H_54_ClN (Aliquat 336) and bis(2,4,4-trimethylpentyl)phosphinic acid (Cyanex 272) [93–95], offering reduced environmental impact compared with conventional solvents due to their negligible volatility and reusability [53,96]. Promising results have been achieved: Riaño *et al*. applied ILE on a used NdFeB magnet (used in electric motors and turbines) and achieved high recovery rates of 99.8%, 99.6% and 99.8% for Co, Nd and Dy, respectively, by utilizing C_32_H_68_NO_3_O for Co and ethylenediaminetertaacetic acid (EDTA) for Nd and Dy [53]. Additionally, Banda *et al*. discovered that ILE extraction with C_33_H_68_NPS resulted in high recovery rates of the REEs, Y and Eu, present in waste fluorescent lamp phosphors [54].

**Table 2.** Recycling technologies for metal recovery from e-waste (laboratory scale).

recycling technique	waste source	processing parameters	metal recovery	ref.
**hydrometallurgy (conventional**)	**initial material**	**solvent, temperature, time, S/L ratio**	**recovery rate (%**)	**ref**.
	PCBs	10 M HNO_3_, 70°C, 5 min; 1:50	>93.4%, Cu, Ag, Nd	[39]
	LCD (mobile phones)	1.0 M H_2_SO_4_, 90°C, 1 h, 1:50	96.4% In	[40]
	LCD (PCU)	HCl:H_2_O (3:2), 80 °C, 1 h, 1:5	60% In	[41]
	lithium-ion battery (LCO from portable device)	EDTA (0.8 M), 90°C, 4 h, 1:50	93.5% Co, 96.4% Li and 97.1% Cu	[42]
	Li-ion battery	C_2_H_4_O_2_ (2 M), 175°C, 2 h, 1:10	Li: 99.9%, Co: 97.6%, Mn: 99.4%, Ni: 98.8%	[43]
	lithium iron phosphate battery	(NH_4_)_2_S_2_O_8,_ 40°C, 1 h, 100 g l^−1^	99.0% Li	[44]
	discarded compact fluorescent lamps	C_2_H_2_O_4_, HNO_3_, 60°C, 20 min, 1:25	>90% Y and Eu	[45]
	waste multi-layer ceramic capacitors	1 M HNO_3_, 1 M HCl or 0.5 M H_2_SO_4,_ 90°C, 30 min, 5 g/l	97% Ni	[46]
	CPU sockets	4 M HCl, 4 h, 100 g/l, 75 g/l	95.73% Au, 96.67% Cu	[47]
**hydrometallurgy (electrochemical**)	**initial material**	**anode/cathode, temperature, time, S/L ratio**	**recovery rate (%**)	**ref**.
	CPU	anode/cathode: Rh-Ti/Ti	95.0% Cu	[48]
	PCBs	anode/cathode: Rh-Ti/Ti, 8 h	94.5% Cu	[49]
	lithium iron phosphate batteries	anode/cathode: Pt-Ti/316 stainless steel, 3 h	99.0% Li, 99.3% Ni, 87.3% Co	[50]
	ceramic capacitors	working, counter and reference electrodes: titanium electrode, platinum plate and Hg/Hg_2_SO_4_	99.02% Pd	[51]
	waste lithium batteries	anode/cathode: waste lithium battery active materials/copper foil, 6 h	99.85% Li, 43.87% Co	[52]
**hydrometallurgy (ionic liquid extraction (ILE**))	**initial material**	**solvent, temperature, time, S/L ratio**	**recovery rate (%**)	**ref**.
	NdFeB magnet	EDTA, C_32_H_68_NO_3_P; 70°C, 2 h	99.8% Co, 99.6% Nd, 99.8% Dy	[53]
	fluorescent lamp phosphor	C_33_H_68_NPS, 30°C, 30 min	98.2% Y 98.7% Eu	[54]
	NdFeB magnet	R4NCy (0.15 M)	98.7% Nd, 99.0% Pr	[55]
	SmCo magnet	Aliquat 336, Cyanex 272, 50°C, 1 h	98.3% Co, 99.4% Sm	[56]
	PCBs	[BSO_3_HPy]OTf, [BSO_3_HMIm]OTf, [BSO_4_HPy]HSO_4_, [BSO_4_HMim]HSO_4_ and [MIm]HSO_4_, 40°C,120 min, 1:5	100% Cu	[57]
	LCD	betainium bis(trifluoromethylsulfonyl)imide ([Hbet][Tf2N]), 90°C, 2 h, 20 g/l	99.75% In	[58]
	SIM cards	[EB-IM][NTf2]	97.90% Au (III)	[59]
**biometallurgy**	**initial material**	**microbe, temperature, time, pulp density**	**recovery rate (%**)	**ref**.
	AMOLED display (smart phone)	*Bacillus foraminis*, 40°C, 12 d, 70 gl^−1^	100% Ag, 56.8% Mo, 41.4% Cu	[60]
	OLED display screen	*Acidithiobacillus ferrooxidans*, 29°C, 15 d, 3 gl^−1^	100% In	[61]
	PCBs (mobile phones)	*Aspergillus niger*, 30°C, 5 d, 1 gl^−1^	96.0% Cu, 82.0% Ni, 100% Zn	[62]
	PCBs (appliances)	*Leptospirillum ferriphilum* and *Sulfobacillus benefaciens*, 35°C, 2 d, 10 gl^−1^	96.0% Cu, 73.0% Ni, 85.0% Zn	[63]
	PCBs (computers)	*Acidithiobacillus ferrivorans*, 23°C, 7 d, 10 gl^−1^	98.4% Cu	[64]
	lithium-ion battery	*Aspergillus niger*, 30°C, 14 d, 10–20 gl^−1^	100% Cu, 100%, 77.0% Li, 75.0% Al, 64.0% Co, 54.0% Ni	[65]
	lithium-ion battery (spent mobile phone)	*Aspergillus nige*r, 30°C, 14 d, 10 gl^−1^	100% Cu, 95.0% Li, 70.0% Mn, 65.0% Al, 45.0% Co, 38.0% Ni	[66]
	lithium-ion battery (laptops)	*Thermophilic bacteria*, 45°C, 48 h, 2.5 gl^−1^	99.9% Co, 99.7% Ni, 84.0% Li	[50]
	LCD panels	*Aspergillus niger*, 30°C, 15 d, 50 g/l	100% In	[67]
	TV circuit boards	*At. ferrooxidans*, *L. ferrooxidans*, *At. thiooxidans*, 35°C, 1−8 g/l	83% Cu	[68]
	spent household batteries	ferrooxidans, 30°C, 10 g/l	87% Ni, 67% Cd, 93.7% Co	[69]
**pyrometallurgy**	**initial material**	**reactor type, temperature, time, condition**	**recovery rate (%**)	**ref**.
	Li-ion battery (slag after crushing)	roasting, 800°C, 2 h, air	73.7% Mn, 73.3% Li	[70]
	Li-ion battery cathode material recovered at 500°C (LiFePO4)	roasting, 200°C, 2 h, air	96.0% Li	[71]
	Li-ion battery (LCO) cathode and anode material	pyrolysis, 1000°C, 0.5 h N_2_ atmosphere	95.7% Co, 98.9% Li	[72]
	Lithium-ion battery cathode (LiCoO_2_) and coal	pyrolysis, 800°C, 10 min, N_2_ atmosphere	96.8% Co, 88.7% Li	[73]
	PCBs	pyrolysis, 800°C, 20 min, air	91.7% Cu	[74]
	PCBs	pyrolysis, 550°C, 120 min, vacuum	99.9% Cu	[75]
	lithium-ion battery cathode (LiNi0.8Co0.1 Mn0.1O2) and anode (graphite)	pyrolysis, 800°C, 180 min, CO_2_ atmosphere	70.0% Ni, 70.0% Co, 60.0% Mn, 99.0% Li	[76]
	waste light-emitting diodes	pyrolysis, 1373 K, 60 min, nitrogen	93.48% Ga, 95.67% In	[77]
	PCB (mobile phones)	pyrolysis 400°C for 30 min	93% Cu, 100% Ni, 100% Zn,100% Pb	[78]
	waste LCD panels	pyrolysis 300°C and 50 Pa, vacuum	99.97% In	[68]
**hybrid recycling strategy**	**initial material**	**method**	**recovery rate (%**)	**ref**.
	Li-ion battery cathode (LCO)	hydro-pyro-electro metallurgy (separate reactor): hydrometallurgy-solvent H_2_SO_4_ and H_2_O_2_, 70°C, 60 min pyrometallurgy-500°C, 10 h, air electrometallurgy-anode/cathode-Ni10Cu11Fe/Co_3_O_4_	99% Li, 99% Co	[79]
	Li-ion battery cathode (LMC)	bio-electro-hydro metallurgy (single reactor): biometallurgy-microbe: sulfur-oxidizing bacteria, 21 d, pulp density 10% electrometallurgy-anode/cathode: graphite/stainless steel hydrometallurgy-solvent: biogenerated H_2_SO_4_	91.5% Co, 93.6% Li, 87.9% Mn	[80]
	PCBs	bio-hydrometallurgy (separate reactor): biometallurgy-microbe: *Leptospirillum ferriphilum*, 72 h hydrometallurgy-solvent: biogenerated Fe_2_(SO_4_)_3_, 200 h	99% Cu	[81]
	Li-ion battery (coin cell)	bio-hydrometallurgy (separate reactor): biometallurgy-microbe: *Acidithiobacillus* *thiooxidans*, 11 d, 30 gl^−1^ hydrometallurgy-solvent: sulfuric acid	99% Li, 60% Co, 20% Mn	[82]
	Gallium (Ga)-bearing waste	oxidation process coupled with NaOH leaching, calcination temperature (600−1400°C) and time (3−12 h), NaOH concentration (0.5−2.5 mol/l), solid-to-liquid ratio (25−150 g/l), leaching temperature (50−90°C) and time (0−240 min)	92.65% Ga	[83]
	LED industry dust	ball milling/leaching: the rotation speed was 150 rpm and the milling time was 24 h. The furnace temperature increased linearly 10°C/min until it reached the requisite temperature (in the range of 800−1200°C), M HCl, 100°C, 20 g/l	73.68% Ga	[84]
	PCBs (mobile phones)	hydrometallurgy + electrochemistry (separate reactor): hydrometallurgy-solvent: 2.0 mol/l HCl electrochemistry: ion exchange using Amberlite XAD−7HP resin	95% Au	[85]
**emerging recycling technologies**	**initial material**	**method**	**product**	**ref**.
microrecycling (thermal micronizing)	PCBs (computer)	first step 500°C, second step 1000°C, inert environment	Cu, Sn	[86]
microrecycling (thermal nanosizing)	ZnC battery	first step 500°C, second step 1000°C, inert environment	Zn, Mn (in oxide form)	[87]
supercritical fluid extraction	PCBs	solvent: water, acetone ethanol, CO_2_; 220–350°C	Cu	[88–90]
supercritical fluid extraction	LCD	solvent: water, CO_2_; 250–400°C	In	
cryo-milling	PCBs	low-temperature ball milling	polymer, oxide and metal	[91]

Despite the significant potential of electrochemical and ILE technologies for economically and environmentally sustainable metal recovery from e-waste, the number of studies reported to date remains limited. Therefore, further research is crucial to fully comprehend their capabilities and broaden their application for a more sustainable and cost-effective e-waste recycling process.

### (c) Biometallurgy

Conventional metal recovery processes, characterized by high energy demands and environmental pollution, have prompted the exploration of sustainable and environmentally friendly alternatives. Biometallurgy emerges as a promising candidate in this pursuit [34]. While exhibiting extended reaction times compared with other metallurgical methods, it offers reduced energy consumption and environmental impact. Notably, although conceptually similar to hydrometallurgy due to its aqueous environments, biometallurgy requires separate discussion due to its distinct mechanisms and limitations.

Several studies have investigated diverse microbial species for their roles in metal extraction (table 2):

- Chemolithoautotrophs (e.g. *Acidithiobacillus ferrooxidans*, *Leptospirillum ferrooxidans*, *Sulfobacillus thermosulfidooxidans*): these energy-efficient microbes utilize inorganic molecules and CO_2_ for growth generating CO_2_ fixation capability [97]. Primarily acidophilic, they oxidize Fe^2+^ and sulfuric compounds, facilitating metal leaching [97].
- Heterotrophic microbes (e.g. *Chromobacterium violaceum*, *Pseudomonas aeruginosa* and *Pseudomonas fluorescence*): relying on organic sources like glycine, these microbes produce cyanide-containing products that enhance metal leaching [98].
- Fungi: these employ three main mechanisms:
  1. Acidolysis: fungi produce acid lixiviants like gluconic, citric and oxalic acids, crucial for metal leaching [34,99].
  2. Complexolysis: stabilizes metal ligands dissolved during acidolysis [100].
  3. Redoxolysis: facilitates metal leaching through electron transfer fungi [101].

Several studies highlight the effectiveness of biometallurgy for e-waste recycling. Ahmadi *et al*. recovered Ag, Mo and Co from smartphone display waste using *Bacillus foramini*s (heterotrophic alkaliphile). Organic acids (C_4_H_6_O_6_, CH_2_O_2_, C_2_H_4_O_2_, C_3_H_6_O_3_, C_6_H_8_O_7_ and C_3_H_6_O_2_) generated from *B. foraminis* functioned as a solvent, achieving recovery rates of 100%, 56.8% and 4.14% for Ag, Mo and Co, respectively [60]. Pourhossein *et al*. achieved a 100% recovery rate from OLED touchscreens using *Acidithiobacillus ferrooxidans* (chemolithoautotroph). Microbial activity generated H_2_SO_4_, facilitating the leaching process [61]. Fang *et al*. recovered Cu, Ni and Zn from waste PCBs with 85.9%, 80.4% and 100% efficiency, respectively, using *Aspergillus niger* (fungus). The fungus generated organic acids like C_6_H_8_O_7_ and C_2_H_2_O_4_, promoting metal leaching [102]. Horeh *et al*. demonstrated the role of organic acids from *A. niger* in metal recovery. They achieved 100% recovery of Cu and Li, and 77%, 75%, 65% and 54% recovery for Mn, Al, Co and Ni from spent Li-ion batteries using acids like C_6_H_8_O_7_, C_2_H_2_O_4_, C_4_H_6_O_5_ and C_6_H_12_O_7_ generated from the fungus [65].

Despite its effectiveness and environmental benefits, biometallurgy faces challenges for practical applications due to:

— Significant sludge generation, which requires proper management.
— Sensitive operating conditions necessitate precise control.
— Long retention time, which can impact efficiency.

Therefore, further research is crucial to enhance the efficiency and effectiveness of the process and increase overall compatibility for practical metal recovery.

There are contradictory results in the literature on the life cycle assessment (LCA) study for the environmental sustainability or the economic feasibility of metal recovery by biometallurgy [103]. For instance, researchers quantitatively assessed the environmental sustainability of the biometallurgy for copper recovery from e-waste and found that it has a significant environmental impact compared with the pyrometallurgical technique [104]. In another techno-economic study, researchers showed that it is economically not feasible for gold recovery from e-waste [105]. On the other hand, many researchers are claiming that the biometallurgical method for metal recovery from e-waste is environmentally sustainable and economically feasible. There is a clear balance in different environmental impact categories and technical pathways for metal recovery by bioleaching.

### (d) Pyrometallurgy

Pyrometallurgy, a widely employed technique in industrial e-waste processing, offers distinct advantages for treating e-waste containing substantial metallic and non-metallic compounds, particularly polymers and metal alloys [106]. However, in the case of metal recycling from e-waste, the non-metallic parts, such as plastics, behave as the reducing agent. At high temperatures, plastics degrade and convert into carbon and reducing gas. Waste material such as waste PCB is rich in Cu and other metals, and is subject to more than 1200°C for copper recovery where the plastics and resins of PCB degrade at less than 450°C and generate CO_2_, CO and solid carbons, etc. [86]. In the case of waste LIBs, the cathode materials of LIBs, such as LiCoO_2_, are reduced by graphite at high temperatures and Co is recovered [36]. The pyrolysis utilizes thermal treatment to convert plastics and other organic compounds into valuable fuels and chemicals and recover metal alloys, oxides, etc., achieving both plastic valorization and metal recovery.

In the pyrometallurgy process, electronic waste is heated in a smelter at high temperatures. It involves numerous methods such as incineration, chlorination, smelting and roasting. Among them, smelting is a powerful and effective method that incorporates different modern equipment such as a Mitsubishi continuous smelter, Outokumpu flash smelting and Noranda reactor system. In a combustion chamber, the process is executed at a very high temperature, i.e. 600 to 1200°C. Inside the combustion chamber sintering processes, different reactions and melting take place [107]. E-waste consists of different alloys, non-metals and metals and the recovery of desired metals from the e-waste through the pyrometallurgy process has become a critical challenge. In the first step, the desired metals are melted at high temperatures, then condensed and in the final step recovered. For metal recovery from e-waste pyrometallurgical technology has been adopted by multiple companies. However, for the pure metal recovery from e-waste after the application of the pyrometallurgy process, secondary technology like the electrometallurgy and hydrometallurgy processes are required for some cases.

Pyrometallurgy encompasses various processes, each with specific applications (table 2):

— Calcination: it eliminates impurities from solid materials under limited oxygen conditions, resulting in high-purity metal alloys [108]. It can also convert metal hydroxides/carbonates into oxides by removing H_2_O/CO_2_, respectively [109].
— Roasting: a gas–solid reaction occurring at high temperatures with O_2_, primarily used to obtain metal oxides from metal sulfides present in e-waste or ore [78].
— Carbothermic reduction: a promising technology involving co-pyrolysis of e-waste and carbon materials [110]. Metal oxides in e-waste are transformed into their reduced forms, while carbon feedstocks become CO/CO_2_ [36].
— Vacuum pyrolysis: under vacuum conditions, the vacuum pyrolysis process is carried out. In terms of apparatus, vacuum pyrolysis is almost similar to the standard pyrolysis process. However, the main difference is that in vacuum pyrolysis, the system is fully sealed and a vacuum pump is utilized. The vacuum pyrolysis process has been used for the recovery of metals from e-waste such as Co recovery from lithium-ion batteries [111], earth elements and Ag recovery from light-emitting diodes [112], Ag, Au and Cu recovery from integrated circuits [113], Sn and Cu recovery from printed circuit boards [114], tantalum alloy recovery from tantalum capacitors [115] and indium recovery from liquid crystal displays [116]. However, the vacuum pyrolysis process cannot recover pure metals singly, it requires assistance from other processes like physical–mechanical separation.
— Catalytic pyrolysis: catalytic pyrolysis of e-waste is considered more effective as it helps to decrease the temperature of degradation. Again because of the presence of catalyst, the composition of the product can be affected more easily [117]. The most commonly utilized catalysts in the pyrolysis process are metal oxides, transition metals and zeolites. From the waste PCBs through the application of catalytic pyrolysis, Fe, Cu and Al were recovered [118]. For the recovery of Cu from waste PCBs, Ni and Fe metals were utilized as catalysts [119]. As biomass contains huge amounts of hydrogen/carbon, so during the co-pyrolysis of e-waste and biomass it can act as a hydrogen donor [120].

Several studies display the effectiveness of pyrometallurgy in e-waste recycling. Hossain *et al*. achieved more than 99% recovery of Co, Cu and Al, respectively, from Li-ion battery slag using a two-step thermal process from 600 to 1400°C [36]. In another study, the researcher recovered Cu and Sn-based alloys from waste PCB with 99.9% efficiency with pyrolysis under inert conditions [121]. Plastics were thermally decomposed into usable carbons and fuels as a co-product from waste flexible PCBs [122]. Hossain *et al*. demonstrated high-purity metal and valuable chemical production from PCB materials through carbothermic reduction and thermal transformation. This approach highlights the potential for a circular economy in e-waste recycling, such as waste PCBs from various electronic devices, spent batteries, etc. While calcination and roasting technologies are established, pyrolysis and carbothermic reduction require further research due to their reduced reliance on chemical agents and generation of valuable gas/liquid products instead of toxic chemicals.

### (e) Combined metallurgical method

Traditional metallurgical techniques for e-waste processing face limitations, including high costs, environmental concerns and inefficiencies. To overcome these drawbacks, combining multiple techniques has emerged (table 2). Biohydrometallurgy utilizes microorganisms as green solvents in hydrometallurgy [123]. Pyro-electrometallurgy facilitates metal phase transformation and accelerates reaction times, enhancing processing and refining efficiency [124].

Hossain *et al*. recovered Li and Co in the form of carbonates from Li-ion battery cathode material (LiCoO_2_) using a hybrid hydrometallurgy–pyrometallurgy–electrometallurgy approach. Li was recovered as lithium carbonate (Li_2_CO_3_) through a solution with sulfuric acid, and Co was extracted as cobalt hydroxide (Co(OH)_2_). This hydroxide was processed by the pyrometallurgical treatment process at 500°C in air to transform it into cobalt oxide (Co_3_O_4_) [125]. In another study, researchers employed an electrochemical process to turn the cobalt oxide into pure cobalt powder [79]. The powder form of extraction has more economical benefits than the oxidized form, thereby increasing the efficacy of the overall recovery process.

Sodha *et al*. extracted Cu from waste PCBs using biohydrometallurgy using biogenerated ferric iron, which acted as a green solvent for copper extraction [81].

Table 3 shows the commercial adoption of different technologies for the metal recovery of e-waste. Pyrometallurgy and mechanical separation are the main techniques commercially adopted by industries, while hydrometallurgy is adopted for metal refining after the reclamation by pyrometallurgical processes or mechanical separation.

**Table 3.** Commercially adopted techniques for metal recovery from e-waste.

commercial technologies	technology	metals recovered	reference
world’s first patent of metal recycling from e-waste, Aleksandrovich’s Patent	pyrometallurgy	platinum group metals and Au	[126]
Ronnskar smelting process, preliminary trial state, Sweden	pyrometallurgy	Cu and precious metals	[127]
Umicore smelting process, fully fledged trial, Belgium	pyrometallurgy	metals from electronics, such as PCBs, LIBs, etc.	[128]
Dowa, Japan	pyrometallurgy	Cu, Au, platinum group metals, Ag, Ga	[129]
Umicore’s metal refining for precious metals, Belgium	pyrometallurgy, hydrometallurgy	base metals, precious metals, PGMs, selenium (Se), tellurium, Indium (In)	[130]
Boliden Rönnskär, Sweden	pyrometallurgy	Cu, Ag, Au, Pd, Ni, Se, Zn, Pb	[131]
Kaldo converter	pyrometallurgy	precious metals like Au and Ag	[132]
Aurubis, Germany		Au, Ag, Cu, Pb, Zn, Sn, PGMs	[133]
Eldan recycling, Spain	mechanical separation	ferrous and stainless steel, non ferrous metals (Al, Cu, brass), refining material containing Cu, brass, Zn, Pb, platinum group metals, etc.	[133]
NEC Group in Japan	mechanical separation and IR heating	Cu-rich product	[133,134]
PCB manufacturing waste recycling, Taiwan	hydrometallurgy, mechanical separation	Cu, Sn, Pb, Zn, etc.	[133]
Sepro urban metal process, Canada	mechanical separation, gravity separation, pyrometallurgy	Cu, fine heavy metal slag	[135]
Shanghai Xinjinqiao Environmental Co., Ltd, and Yangzhou Ningda Precious Metal Co., Ltd, China	vacuum pyrometallurgy	Cu, Pd-Bi alloy, Cd, Zn	[136]
SwissRTec AG	mechanical separation	Cu, Al, steel	[137]
WEEE Metallica, France	mechanical separation, eddy current separation	Fe, Al, etc.	[138]

### (f) Emerging technologies for e-waste recycling

Among the emerging technologies, supercritical fluid technology is promising for its environmental sustainability in terms of effectively removing the brominated compounds and the organic elements and recovering the metals, such as Cu [88], Au [139], In [140], Ta [106], etc. Nevertheless, the cost-effectiveness is not satisfactory and the high investment cost makes it difficult to implement for metal manufacturing. To make the technologies and processes economically feasible and environmentally sustainable, researchers have proposed small-scale recycling technologies that are called microrecycling technology [3]. Microrecycling is a recycling technology where e-waste, such as PCBs, spent batteries, hard drives and LCDs, undergo high-temperature thermal transformations. The recycling strategies are judiciously selected to be implemented into the small volume of waste locally, such as a few kilograms to several hundreds of grams so that the waste does not need to be transported to the centralized places where the bigger facilities are situated. This approach saves transportation costs and creates a local supply chain of the metals, which can contribute to the local economy and new jobs locally [9].

Researchers from the Sustainable Material Research and Technology Centre (SMaRT) have successfully employed various microrecycling techniques. Selective thermal transformation techniques have been used to recycle multi-material waste like e-waste to extract copper-based and copper tin-based alloys [121,141]. Similarly, e-waste like NiMH batteries has been processed using microrecycling techniques to extract nickel-based alloys [142], as well as lithium and cobalt alloys from LIB batteries [78]. Another noteworthy accomplishment involves the utilization of oxidation-reduction processes in microrecycling to extract REEs from e-waste, achieving a remarkable recovery rate of 90–100% [143]. In this research, the anode part of nickel metal hydride batteries was subject to oxidation in air at 1000°C, and then reduction was carried out at 1550°C. Oxides of Co and Ni were diffused in iron, transformed to ferronickel alloy and REEs (i.e. La, Ce, Nd and Pr) were separated as oxides. These advancements underscore the potential and versatility of microrecycling methods in addressing diverse recycling challenges.

### (g) LCA studies

LCA studies, with some scenarios, have been presented to assess the environmental implications associated with e-waste, covering the entire life cycle from production to end-of-life [144]. These investigations have scrutinized and compared both the environmental and economic dimensions of various e-waste management scenarios, encompassing direct disposal, end-of-life disposal methods such as landfilling and incineration, and value-recovery strategies.

In a study by Hong *et al*., the environmental impact of e-waste disposal processes was evaluated, considering both direct disposal (ND) and end-of-life processes (ELD), inclusive of landfilling and incineration [145]. Their findings indicated that the overall environmental impacts were considerably higher in the direct disposal scenario compared with the end-of-life disposal scenario. The results of the LCA midpoint assessment of the ND scenario for the categories of human toxicity (84.4 kg 1,4 DB eq.), terrestrial ecotoxicity (37.8 kg 1,4 DB eq.), freshwater ecotoxicity (4.39 kg 1,4 DB eq.) and marine ecotoxicity (2.25 kg 1,4 DB eq.) were evidently higher than those under the ELD scenario with human toxicity (4.15 kg 1,4 DB eq.), terrestrial ecotoxicity (1.08 × 10^−3^ kg 1,4 DB eq.), freshwater ecotoxicity (8.67 × 10^−2^ kg 1,4 DB eq.) and marine ecotoxicity (8.90 × 10^−2^ kg 1,4 DB eq.).

In contrast to the ELD scenario, relying on the incineration process, it exhibited the highest environmental impacts concerning climate change, ozone depletion, urban land occupation, land transformation and water depletion. The elevated impact is attributed to the generation of greenhouse gases and reliance on coal-based electricity. Consequently, there is a pressing need to establish an efficient e-waste valorization platform to mitigate the reliance on chemicals and coal. Li *et al*. assessed the environmental impacts of e-waste recycling using LCA [146], which demonstrated that traditional e-waste recycling is effective in tackling issues associated with electronic waste. However, they highlighted that to improve the aggregate sustainability of the process, the reduction of energy usage and chemical reagents is necessary.

In addressing these hurdles, Debnath *et al*. conducted a comprehensive LCA study comparing two scenarios: one focusing on standard metal recovery, including eddy current separation, and the other utilizing pyrolysis [147]. The study highlighted that the strategic utilization and commercialization of pyrolysis by-products, such as fuel, chemicals and char, could substantially reduce costs and alleviate environmental burdens associated with energy and chemical consumption.

Although these studies have demonstrated significant environmental advantages of value-recovery from e-waste, relevant research in this area is still lacking. Therefore, further comprehensive research on LCA is required to explore and evaluate the latest advancements in e-waste metal recovery technologies, improve our understanding of the potential benefits and limitations and ultimately construct a sustainable e-waste metal recovery platform.

## 4. Other influencing factors for metal recycling of e-waste

### (a) Green product designs

The implementation of green design principles in electronic products constitutes a pivotal strategy to proactively mitigate e-waste at its origin. This approach emphasizes the integration of environmentally conscious practices throughout the entire life cycle of the product. In the realm of electronic product design, the incorporation of modular, integrated and intelligent technologies, alongside the utilization of high-powered, lightweight and ecologically sound materials, becomes imperative [9]. Strategic design considerations involve the crafting of electronic products that are non-hazardous, energy-efficient, eco-friendly and possess attributes that render them easily recyclable, all while maintaining high reliability and extended lifespan [1,148]. Producers bear a responsibility not only for the creation of such green products but also for facilitating the recycling and secure disposal of electronic devices. Furthermore, there is growing emphasis on encouraging producers to implement green design and production practices, aiming to maximize the life cycle of electronic products and consequently contribute to e-waste reduction [149].

The foundational elements of green design for electronic products encompass design principles, materials selection and construction techniques. Design principles require a comprehensive consideration of environmental attributes such as disassembly, recyclability, maintainability and reusability, streamlining the subsequent recycling process for decommissioned electronic products. The selection of eco-friendly materials with a focus on recyclability is pivotal, explicitly prohibiting the use of toxic and hazardous substances and promoting material reduction for ease of recycling. Construction techniques play a crucial role, as the development of green recycling processes aims to prevent the release of heavy metals and organic substances during electronic waste dismantling. Simultaneously, it seeks to minimize the use of acidic, alkaline and corrosive reagents and chemicals.

### (b) Advances in collection

In response to the evolving landscape of electronic products driven by changing societal needs and continuous product upgrades, the recycling of electronic products faces intricate technical challenges, emphasizing the crucial aspects of collection, disassembly and fine separation. To meet these challenges, a transformative approach leverages the rapid development of the internet and proposes the establishment of an e-waste recycling network cantered around the Internet of Things (IoT) as a key information flow [150].

An innovative solution involves the creation of an integrated e-waste collection system with online/offline integration [151]. For example, Liu *et al*. [150] proposed that in this system, e-waste information is coded for efficient tracking and monitoring, requiring manufacturers to encode electronic products, sellers to register products with identity information and purchasers to submit waste product information on a government-provided third-party platform. Disposers offer online services, facilitate offline transfers and derive profits from recycling, while online supervision and offline financial subsidies form a symbiotic relationship, enabling a ‘closed loop’ management system for waste electrical and electronic products. This streamlined approach addresses technical challenges and establishes an efficient, transparent system aligning with the dynamic landscape of electronic product recycling.

### (c) Recycling of all components

Addressing the escalating complexity of electronic product structure requires a transition from a single technology to a comprehensive approach to achieve effective separation of the e-waste product. The current inadequacies in recycling systems lead to substantial losses of valuable raw materials, contributing to resource wastage [3]. To rectify this, an imperative shift towards advanced technologies is needed, where metals and non-metals will be targeted to recover simultaneously from the e-waste without any loss. It is important to distinguish the reusable and unusable elements in electronic products. The reusable components could be refurbished and reused. The strategy is needed for the targeted recycling, which is fit for purpose, for example, utilizing selective thermal transformation for recovering metals with carbon, oil and gas co-products.

### (d) Right scaled technology implementation

Right scaled technology is needed to tackle the problematic e-waste. In e-waste, the volumes are less compared with the value, and different multi-materials, such as metals, polymers and ceramics, are embedded together in the same small component. So, in most cases, it is not possible to implement conventional metal recycling processes where similar types of scrap materials have huge volumes, such as steel from automotive waste. A modular, decentralized and scalable form of recycling unit is needed that can be deployed in varied scale configurations, implementing tailored recycling technology based on the type of waste generated in the area. These small-scale facilities and decentralization help in a distributed manufacturing system thereby eliminating the need for having large collection centres and the transportation and logistics behind the central recycling facilities. For example, researchers invented small scaled MICROfactorie™ to recycle e-waste using the concept of microrecycling, which refers to the synthesis of value-added materials from small amounts of waste [156]. The recycling technology design needs to be simple, yet effective to liberate value-added materials from waste resources. This technique exhibits adaptability to evolving product designs and material compositions, presenting a facile implementation process with reduced resource demands and economic pressure. This distributed approach with the flexibility of technology could contribute to the creation of a sustainable economic ecosystem where material-flow loops can be effectively closed.

## 5. Conclusions and future research requirements

The rapid increase in worldwide e-waste generation is a direct consequence of the expansive growth in the electronics industry and the swift transition in the market trends for electronic devices. Unfortunately, inadequate management practices, notably landfilling and incineration, exacerbate environmental contamination by releasing toxic chemicals and heavy metals into ecosystems. Recognizing this issue, researchers have diligently explored metallurgical and thermochemical processes to establish a comprehensive value chain, incorporating the recovery of both metallic and non-metallic compounds from e-waste.

Several investigations have demonstrated the viability of reclaiming metal compounds utilizing metallurgical methods like hydrometallurgy, biometallurgy, pyrometallurgy, combined metallurgy, and a few other emerging technologies, such as supercritical fluid extractions, cryomilling and microrecycling techniques, etc. Considering the consistent rise in the yearly market value of metals, approaches for recovering metals and their alloys from e-waste arise as potentially efficient strategies capable of yielding profits and curbing environmental pollution. Consequently, the findings along with emerging technologies, such as implementing a small-scale recycling strategy, smart collection of e-waste, green design strategy, etc., propose an efficient pathway to establish circular economic platforms for electrical and electronics devices. Despite the promising outlook, the practical implementation of these technologies encounters several technical challenges that require careful consideration and resolution for successful integration into waste management systems. Contemporary metallurgical practices and studies that are aimed at reducing energy and chemical consumption should be explored, thereby enhancing overall efficiency, effectiveness and practical applicability. Incorporating the advanced LCA studies that will include the advancements in metallurgy and thermochemical processes is needed. By addressing these key issues, future research endeavours can contribute significantly to the development and implementation of sustainable practices in managing and recycling e-waste for valuable metals to ensure the circular economy of valuable metals.

## Data Availability

Supplementary material is available online [152].
